# A cross-modal investigation of the neural substrates for ongoing cognition

**DOI:** 10.3389/fpsyg.2014.00945

**Published:** 2014-08-26

**Authors:** Megan Wang, Biyu J. He

**Affiliations:** National Institute of Neurological Disorders and Stroke, National Institutes of HealthBethesda, MD, USA

**Keywords:** ongoing cognition, semantic processing, linguistic processing, cross-modal, default-mode network, fMRI, conscious flow

## Abstract

What neural mechanisms underlie the seamless flow of our waking consciousness? A necessary albeit insufficient condition for such neural mechanisms is that they should be consistently modulated across time were a segment of the conscious stream to be repeated twice. In this study, we experimentally manipulated the content of a story followed by subjects during functional magnetic resonance imaging (fMRI) independently from the modality of sensory input (as visual text or auditory speech) as well as attentional focus. We then extracted brain activity patterns consistently modulated across subjects by the evolving content of the story regardless of whether it was presented visually or auditorily. Specifically, in one experiment we presented the same story to different subjects via either auditory or visual modality. In a second experiment, we presented two different stories simultaneously, one auditorily, one visually, and manipulated the subjects' attentional focus. This experimental design allowed us to dissociate brain activities underlying modality-specific sensory processing from modality-independent story processing. We uncovered a network of brain regions consistently modulated by the evolving content of a story regardless of the sensory modality used for stimulus input, including the superior temporal sulcus/gyrus (STS/STG), the inferior frontal gyrus (IFG), the posterior cingulate cortex (PCC), the medial frontal cortex (MFC), the temporal pole (TP), and the temporoparietal junction (TPJ). Many of these regions have previously been implicated in semantic processing. Interestingly, different stories elicited similar brain activity patterns, but with subtle differences potentially attributable to varying degrees of emotional valence and self-relevance.

## Introduction

Imagine you are at a New Year's party. A friend is recounting her recent trip to New Zealand, meanwhile the television in front of you is playing a tennis match. You find yourself staring at the TV without following the game, and fully absorbed in your friend's exciting story. Alternatively, if you are a tennis fan, you might find yourself following the game and missing part of your friend's story, recognizing the need to correct your attentional focus. In both cases, the sensory inputs to your brain are identical while the stream of your conscious content is rather different. What brain mechanisms might contribute to the ongoing flow of your conscious mind beyond sensory inputs (James, 1890; Dehaene and Sigman, 2012)?

One initial approach to investigating this question is to identify brain areas reliably modulated by similar content of conscious flow in the face of changing sensory inputs. Since it cannot be excluded that certain unconscious processes are also consistently modulated across time in such a paradigm, consistent modulation in the face of similar conscious stream constitutes a necessary albeit insufficient condition for identifying brain activities potentially underlying internal conscious flow. Alternatively, reliable modulation by the same sensory inputs in the presence of different conscious content, as in the example above, would reveal lower-level processing of sensory stimulus. Because both the sensory stimulus and the conscious flow in this example are time-varying features, “reliable modulation” means a similar temporal response profile across time. The approach of using reliable modulation by naturalistic stimuli to probe a brain region's involvement in stimulus processing has been successfully applied to both within- and across- subject analyses (Hasson et al., 2010). We extended this approach to studying brain areas consistently modulated by the evolving content of a story independent of the modality of sensory input, by presenting subjects with auditory speech or visual text. Because listening to or reading a story would not only trigger linguistic processing, but also post-linguistic processes such as imagery, theory-of-mind, episodic and emotional processing, we hereafter refer to the totality of these processes “*ongoing cognition.*” Importantly, these processes may include brain activities directly underlying the conscious flow as well as their prerequisites and consequences (Bachmann, 2009; Aru et al., 2012; de Graaf et al., 2012; Li et al., 2014).

Many previous studies have studied the convergence of neuroanatomy for the processing of spoken and written languages (Chee et al., 1999; Calvert, 2001; Spitsyna et al., 2006; Jobard et al., 2007; Lindenberg and Scheef, 2007). These previous studies have generally assessed the activation magnitude of a brain region in response to spoken or written language, upon which convergence (i.e., activation in both tasks) was determined. Alternatively, an interaction effect is sometimes determined in multisensory integration studies (Raij et al., 2000; Calvert, 2001; van Atteveldt et al., 2004), which quantifies the activation magnitude to the simultaneous presentation of both modalities beyond the sum of activation magnitudes to each modality presented alone. However, the measure of activation magnitude provides only a crude estimation of a brain region's involvement in the task. For example, a brain region can be activated in both the auditory and visual tasks but with distinct temporal modulation profiles, which would indicate different kinds of processing in the two tasks. By contrast, reliable cross-modal modulation of the temporal response profile of a brain region during an evolving story presented as visual text or auditory speech would constitute stronger evidence for its involvement in the ongoing cognition elicited by the story.

We conducted two experiments. In the first experiment, we presented a story (“Cage”) to different subjects via either visual or auditory presentation. Specifically, the visual and auditory presentations were controlled to advance at roughly the same speed. We identified the brain regions exhibiting similar time courses across these two subject groups. Because the sensory inputs are presented through different modalities but the story content is the same, these brain regions are expected to underlie modality-invariant linguistic and post-linguistic processes. In the second experiment, we presented two different stories (“Fish” and “King”) simultaneously to the subjects, one auditorily, one visually. In different functional magnetic resonance imaging (fMRI) runs, the subject was cued to pay attention to one modality vs. the other, thus following different stories. Correlating brain activities during an identical task condition across subjects allowed us to identify brain regions consistently modulated by this task condition. By contrast, correlating brain activities between task conditions that had identical physical stimuli but different attended sensory modalities (and thus different stories) allowed us to extract brain activities modulated purely by the sensory inputs.

## Materials and methods

### Subjects

Twenty-seven healthy right-handed English-fluent subjects between 19 and 38 years of age (8 males) with normal or corrected-to-normal vision participated in the study. All subjects provided written informed consent. The experiment was approved by the Institutional Review Board of the National Institute of Neurological Disorders and Stroke. Seven subjects were excluded due to excess movement in the scanner, and two additional subjects were excluded due to failed registration to the atlas. Thus, eighteen subjects (6 males) were included in the analysis.

### Stimuli and task design

Three short narratives, referred to as the “Cage,” “King” and “Fish” stories, were presented visually and auditorily. “Cage” was compiled from the Wikipedia entry on John Cage's composition *4′33″* and was used in Experiment 1. “King” is the short story “The Three Questions” by Leo Tolstoy and “Fish” is a short story called “Fred's Fish”; they were used in Experiment 2. Complete transcripts for the three stories and the experimental stimuli used in all conditions can be found in Supplementary Materials. These stories were chosen as materials that most subjects would not be familiar with, to ensure that subjects would have to attend carefully to comprehend the stories. For the auditory version of these stories, a female native English speaker recorded each story with a Logitech H530 headset and edited the recording using Audacity 1.3.13-beta (e.g., by removing breathing artifacts). The visual version was presented in subtitles format using Aegisub 2.1.8, such that each phrase was on screen for the same duration as it was spoken in the auditory version. During the pauses between sentences, a cross-hair was presented at the center of the screen such that the screen was never blank. In Experiment 2, the visual and auditory stories, which had the same duration, were combined in MeGUI to create a stimulus consisting of “Fish” story subtitles simultaneously presented with the “King” story audio recording (FishV+KingA), and a second stimulus consisting of “King” story subtitles simultaneously presented with the “Fish” story audio recording (KingV+FishA).

**Figure 1:**
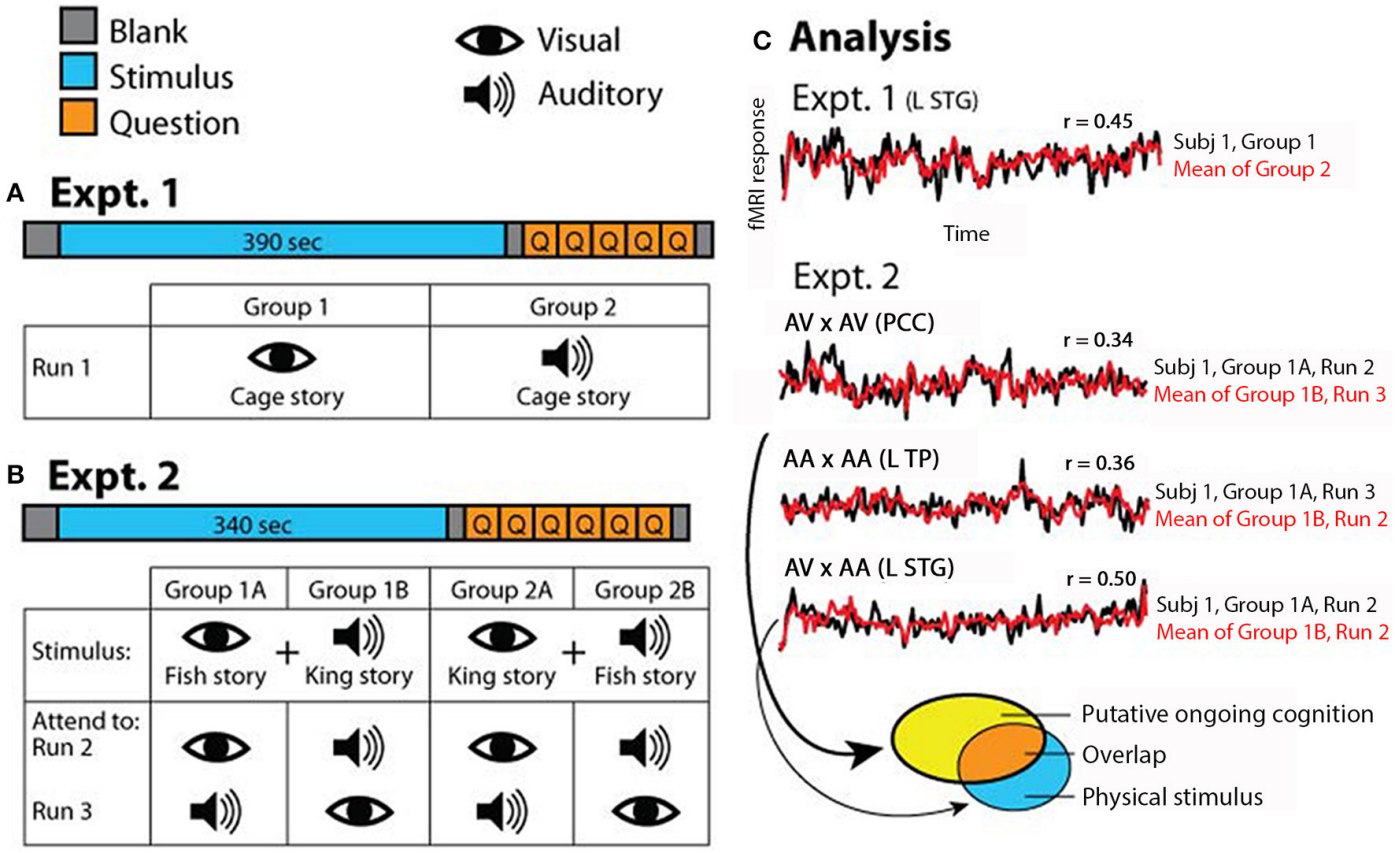
**Experimental paradigm. (A)** In Experiment 1, which consisted of one fMRI run, half of the subjects read the “Cage” story and the other half listened to the “Cage” story. **(B)** In Experiment 2, which consisted of two runs, half of the subjects were exposed to simultaneous auditory recording of “King” story and visual text presentation of “Fish” story (Group 1), and the other half exposed to simultaneous auditory recording of “Fish” story and visual text presentation of “King” story (Group 2). Subjects were instructed to attend to a specific sensory stream in one fMRI run and the other stream in the second run; the order of attended modality was counterbalanced within each group. **(C)** Schematic of analysis approach. In Experiment 1, correlations were computed between the two groups of subjects for each voxel (results shown in Figure 2). In Experiment 2, correlations were computed for each voxel between subgroups of subjects under identical task condition (AV × AV or AA × AA), or identical stimulus condition but opposite attentional focus (AV × AA) (results shown in Figure 3). AV, attend to visual; AA, attend to auditory. In all figures, symbol “×” means correlation. The brain regions from which the example time courses (over the entire stimulus period) were extracted are indicated in the graph. L STG, left superior temporal gyrus; PCC, posterior cingulate cortex; L TP, left temporal pole. The ellipses at the bottom illustrate the analysis depicted in Figure 4, with each ellipse indicating the significant brain areas from an analysis. For detailed methods see Materials and Methods.

In Experiment 1, half of the subjects (*N* = 9) followed the “Cage” story presented visually while the other half (*N* = 9) followed the “Cage” story presented auditorily during fMRI scan (Figure 1A). In the visual condition, subjects read the story presented phrase-by-phrase at the center of the screen. In the auditory condition, subjects listened to the story presented via headphones (Avotec Inc., FL) while looking at a blank screen (visual fixation was not required). The detailed structure of Experiment 1 is as follows: 10 s of blank, 390 s of stimulus, 5 s of blank, 5 multiple-choice questions probing the comprehension of the story presented for 10 s each, and finally 5 s of blank (Figure 1A). Subjects were asked to answer each question during the 10-s interval using one of four buttons. Occasionally subjects pressed outside the allotted time interval or pressed answers twice; in those cases, answers were confirmed verbally immediately after the run ended.

In Experiment 2, subjects were divided into two stimulus groups, and instructed to attend to one modality in one fMRI run and the other modality in the second fMRI run, in a counter-balanced manner. Half of the subjects (*N* = 9) were presented the FishV+KingA stimulus, with five of them instructed to attend to the visual story (“Fish”) in the first run and then to the auditory story (“King”) in the second run, and four subjects instructed with the opposite order (Figure 1B, Group 1). The remaining nine subjects were presented with the KingV+FishA stimulus, again with the attending sequence counter-balanced across subjects (Figure 1B, Group 2). In the “Attend to Visual” (AV) condition, subjects were told to ignore the auditory input and focus on reading the story presented at the center of the screen. In the “Attend to Auditory” (AA) condition, subjects were instructed to focus on listening to the story presented auditorily, and to maintain fixation at the center of the screen but ignore the story presented visually.

Each of the two runs in Experiment 2 contained 10 s of blank, 340 s of stimulus, 5 s of blank, 1 question on intrusion presented for 10 s, 5 multiple-choice comprehension questions presented for 10 s each, and finally 5 s of blank (Figure 1B). The intrusion question asked the subject to rate the level of intrusion by the unattended story, from 1 (no intrusion) to 4 (full intrusion, or followed both stories equally). Subjects answered the question using one of four buttons. Comprehension questions tested the comprehension of the attended story only, to ensure that the subject was following instructions by attending to the correct sensory stream.

### Eye tracking

Subjects' eyes were monitored throughout the entire experiment using an MR-compatible eye tracker (NordicNeuroLab Inc., WI) through which the visual stimulus was presented. The eye tracker was calibrated at the beginning and end of the experiment, and more frequently as needed if there was excess head movement. We monitored the subjects' eyes closely to ensure that they were following instructions. That is, the subjects' gaze fixated on the center of the screen when instructed to attend to the auditory stream, and they were seen to make quick saccades when instructed to attend to the visual stream.

### fMRI data acquisition

Functional and anatomical MRI was conducted on a General Electric 3T scanner with an 8-channel head coil. Anatomical images were obtained using a sagittal magnetization-prepared rapid-acquisition gradient echo (MP-RAGE) sequence with a resolution of 1 × 1 × 1 mm^3^. An axial T2-weighted structural scan was acquired with *TR* = 4200 ms, *TE* = 120 ms and a resolution of 3 × 3 × 3 mm^3^. BOLD-contrast functional images were obtained using a single-shot gradient echo sequence with 39 contiguous transverse slices covering the whole brain (slice thickness = 3 mm, in-plane resolution: 3 × 3 mm^2^, *TR* = 2000 ms, *TE* = 27 ms, flip angle = 90°).

### fMRI data preprocessing

fMRI data were preprocessed as follows: (1) compensation of systematic, slice-dependent time shifts; (2) elimination of systematic odd-even slice intensity difference due to interleaved acquisition; (3) rigid body correction for inter-frame head motion within and across runs; and (4) intensity scaling to yield a whole-brain mode value of 1000 (with a single scaling factor for all voxels). Atlas registration was achieved by computing affine transforms connecting the fMRI run first frame (averaged over all runs after cross-run realignment) with the T2- and T1-weighted structural images. Our atlas representative template included MP-RAGE data from 12 normal individuals and was made to conform to the 1988 Talairach atlas (Talairach and Tournoux, 1988). Data were resampled to 3 × 3 × 3 mm^3^ voxels after atlas registration.

fMRI signals from each run were detrended and the effect of head motion and its temporal derivative were removed by linear regression. We further removed the effect of low-level physical attributes of the stimuli to avoid inter-subject correlations driven by low-level transients in the visual and auditory stimuli, similar to the method used in Honey et al. (2012a). To this end, we determined the transients in the visual stimuli and the sound envelope of the auditory stimuli. For visual transients, the phrase-to-fixation transitions, fixation-to-phrase transitions and phrase-to-phrase transitions were each modeled as a series of delta functions. For the sound envelope, the audio signal was bandpassed between 4 and 4000 Hz and the envelope was extracted using a Hilbert transform. These four regressors (three types of visual transients and the sound envelope) were each convolved with the hemodynamic response function, down-sampled to the sampling rate of the fMRI signal (*TR* = 2 s), and removed from the fMRI data via linear regression. Finally, data from each subject were spatially smoothed with a Gaussian kernel (FWHM = 6 mm).

### Within- and across- modality response reliability

Similar to previous studies (Hasson et al., 2004; Honey et al., 2012a), we assessed the correlations of the fMRI signals during stimulus presentation (length: 390 s in Experiment 1; 340 s in Experiment 2) across subjects at each voxel. The first 5 frames of each fMRI run corresponded to the blank period and were not included in the correlation. Thus, scanner magnetic stabilization was already reached by the beginning of stimulus presentation. In Experiment 1, for within-condition reliability assessment, Groups 1 and 2 (see Figure 1A) were analyzed separately. fMRI signal time course from each subject was correlated with the mean time course across the remaining subjects in the same group. The Pearson correlation r values were transformed into Fisher *z*-values, which are approximately normally distributed. The Fisher z maps were then averaged across all subjects in each group (*N* = 9) to yield the population average. For across-condition reliability assessment, fMRI signal correlations were evaluated between Groups 1 and 2 that followed the same story presented via visual and auditory modality, respectively (Figure 1A). Each subject's fMRI signal time course was correlated with the average time course from the other group. The Pearson correlation r values were transformed into Fisher *z*-values, which were then averaged across all subjects in both groups (*N* = 18) to yield the population average.

In Experiment 2, the correlations were carried out across subjects presented with identical physical stimuli (i.e., between Groups 1A and 1B, and between Groups 2A and 2B, see Figure 1B). Two analyses were carried out. In the first, we correlated fMRI runs in which one subgroup attended to the visual stream and the other subgroup attended to the auditory stream (e.g., Run 2 of both Groups 1A and 1B). To avoid repetition suppression effect (Grill-Spector and Malach, 2001), only the fMRI runs in which the stimulus was presented for the first time were used (Run 2 in Figure 1B). Because the correlation was carried out between subjects presented with identical physical stimulus but attending to different sensory modalities and thus different stories, the brain regions showing reliable responses should be those involved in low-level sensory processing. In the second analysis, we correlated fMRI runs in which the two subgroups of subjects were presented with identical stimulus and attended to the same sensory stream (e.g., Run 2 from Group 1A and Run 3 from Group 1B), which assessed which brain regions were consistently modulated by each task condition. In both analyses, the fMRI signal time course from each subject was correlated with the average time course from the other subgroup. The Fisher-z-transformed correlation maps were averaged across all subjects in each group (*N* = 9, since Groups 1 and 2 were analyzed separately).

### Bootstrapping by phase-randomization to assess significance

We assessed statistical significance using a bootstrapping procedure based on phase-randomization. For each voxel, we applied Fourier transform on the time series, randomized the phase component, and inverted the Fourier transform to obtain the shuffled time series. For Experiment 1, each subject's time series was phase-shuffled and correlated with the original average of the other group; this was done 50 times per subject to create a distribution of 900 bootstrapped correlations. For Experiment 2, bootstrapping was performed 100 times per subject, again to yield a distribution of 900 bootstrapped correlations for each analysis (Groups 1 and 2 were analyzed separately). All of the bootstrap correlations were transformed into Fisher *z*-values. We then calculated the mean and standard deviation (SD) across the distribution of 900 bootstrap iterations. Because in the original analysis the correlations were averaged across subjects, the SD of the bootstrap distribution was corrected by a factor of $\sqrt{N}$, where *N* = 18 in Experiment 1 and *N* = 9 in Experiment 2. The mean of the bootstrap distribution and the corrected SD were then used to convert the original population-average Fisher-z maps into Z-scores, from which statistical significance was determined. To correct for multiple comparisons, we adopted the Monte Carlo method for family-wise error (FWE) correction (McAvoy et al., 2001) and applied a threshold of Z score >3 and cluster size >17 voxels, yielding clusters that survived *p* < 0.05.

## Results

To investigate ongoing cognition using controlled semantic content, we presented stories as auditory speech and/or visual text to subjects and correlated the fMRI time series across subjects to map brain areas that responded reliably to a task condition or across different conditions. We applied this correlational approach to identify brain regions underlying sensory processing vis-à-vis ongoing cognition.

### Behavioral results

For the “Cage,” “King,” and “Fish” stories, subjects correctly answered an average of 4.4 ± 0.17 (mean ± s.e.m. across 18 subjects), 4.2 ± 0.23 and 4.3 ± 0.16 comprehension questions, respectively, and there was no significant difference between stories (*p* = 0.73, Kruskal-Wallis test). In Experiment 1, the level of comprehension was not significantly different between subjects who heard the story and those who read the story (*p* = 0.09, Wilcoxon rank-sum test). In Experiment 2, there was no significant effect of the attended modality (*p* = 0.26, Wilcoxon signed-rank test) or run order (*p* = 0.97) on the level of comprehension of the attended story.

In Experiment 2, we asked an additional question concerning the level of intrusion by the unattended story. The intrusion level averaged across all runs from all subjects was 2.1 ± 0.13. It was not significantly different between the AV and AA conditions (*p* = 0.49, Wilcoxon signed-rank test), or between the first and second fMRI runs (*p* = 0.38, Wilcoxon signed-rank test). Interestingly, when attending to the “King” and “Fish” stories, subjects reported an average intrusion level of 2.5 ± 0.15 and 1.8 ± 0.19, respectively (*p* = 0.01, Wilcoxon signed-rank test), suggesting that the “Fish” story was more intrusive. This is likely due to the fact that the “Fish” story was told in first-person perspective and had more emotional and personal content while the “King” story was a fable told in third-person perspective.

### Experiment 1—“cage” story presented alone via visual or auditory modality

**Figure 2:**
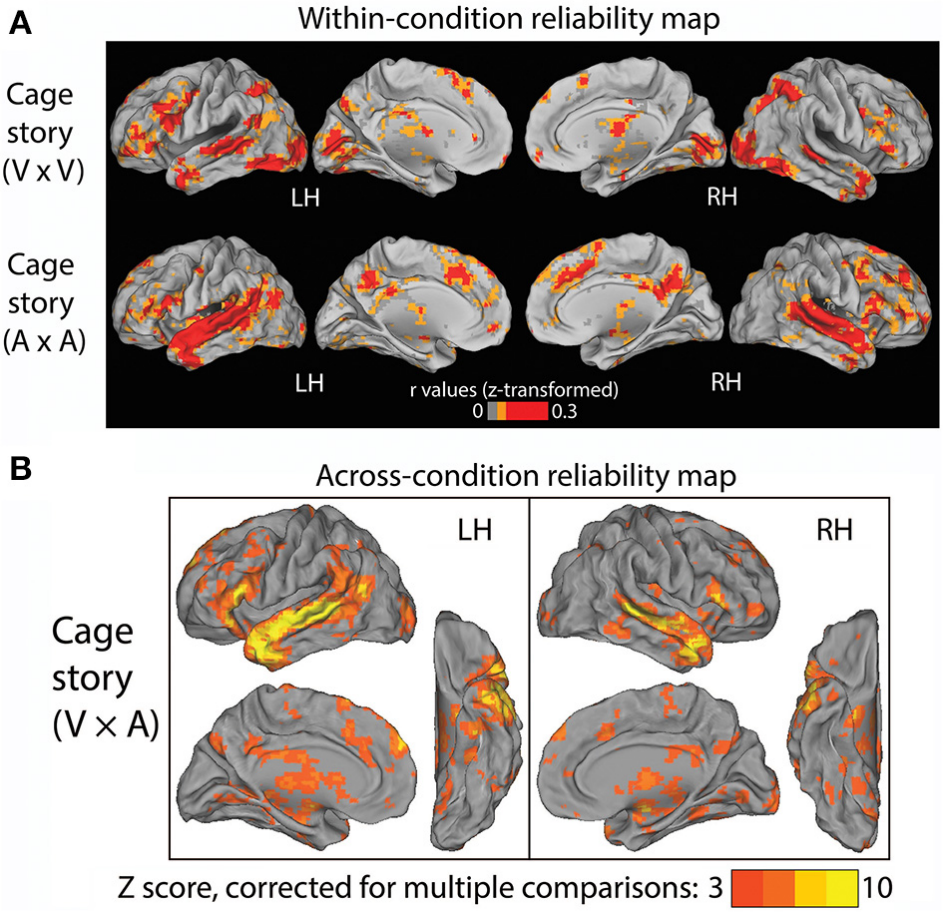
**Results of Experiment 1. (A)** Within-condition reliability map. fMRI time series were correlated between subjects who read the “Cage” story (top row) and between those who listened to the “Cage” story (bottom row). The Pearson correlation coefficients were z-transformed and averaged across all subjects in each group (*N* = 9). **(B)** Across-condition reliability map. fMRI time series were correlated between subjects who listened to the “Cage” story and those who read the same story (V × A, see Figure 1C, Experiment 1). The population-average z-transformed correlation values were compared to phase-shuffled data to determine the *Z*-score (thresholded at *Z* >3, cluster size >17 voxels, corresponding to *p* < 0.05 after correction for multiple comparisons).

In Experiment 1, one group of subjects (*N* = 9) listened to the “Cage” story through headphones, and another group of subjects (*N* = 9) read the “Cage” story presented visually at the center of the screen. The auditory and visual versions of the story were presented at roughly the same speed. First, we identified brain regions that were reliably modulated across subjects within each condition alone. To this end, we computed inter-subject correlations for each voxel within the first group of subjects that read the “Cage” story (Figure 2A, top row), and within the second group of subjects that listened to the “Cage” story (Figure 2A, bottom row). Unsurprisingly, in the reading (“V”) condition, the occipital visual cortices, as well as the intraparietal sulci (IPS) involved in visuospatial attention, are reliably modulated. By contrast, in the listening (“A”) condition, there was extensive reliable modulation of the early and higher-order auditory cortices along the superior temporal gyrus (STG). Both the reading and listening conditions consistently modulated the inferior frontal gyrus (IFG), temporal pole (TP), the superior temporal sulcus (STS), anterior cingulate cortex (ACC), and the thalami. Interestingly, the posterior cingulate cortex (PCC) was substantially more involved in the listening condition than the reading condition.

The above results reveal a common set of brain regions that are reliably modulated in both reading and listening conditions. Nonetheless, it remains unknown whether their temporal response profiles are similar across these two conditions. To address this question, we correlated the fMRI time series across these two groups of subjects to extract brain regions reliably modulated by the “Cage” story regardless of the sensory modality used for stimulus input. The results are shown in Figure 2B (*p* < 0.05, FWE corrected), which included bilateral STS/STG, TP, and IFG, the left temporal parietal junction (TPJ), the dorsal medial prefrontal cortex (dmPFC) and the thalamus. There was a slight left asymmetry in the response pattern.

### Experiment 2—“fish” and “king” stories presented simultaneously via auditory and visual modalities

In Experiment 2, one group of subjects (*N* = 9) were simultaneously presented with the “Fish” story as visual text and the “King” story as auditory speech (FishV+KingA), and instructed to attend to the two sensory streams in alternate runs in a counter-balanced manner (Group 1 in Figure 1B). A second group of subjects (*N* = 9) were presented with the “King” story as visual text and the “Fish” story as auditory speech (KingV+FishA) and also instructed to attend to different sensory streams in alternate runs (Group 2 in Figure 1B).

**Figure 3:**
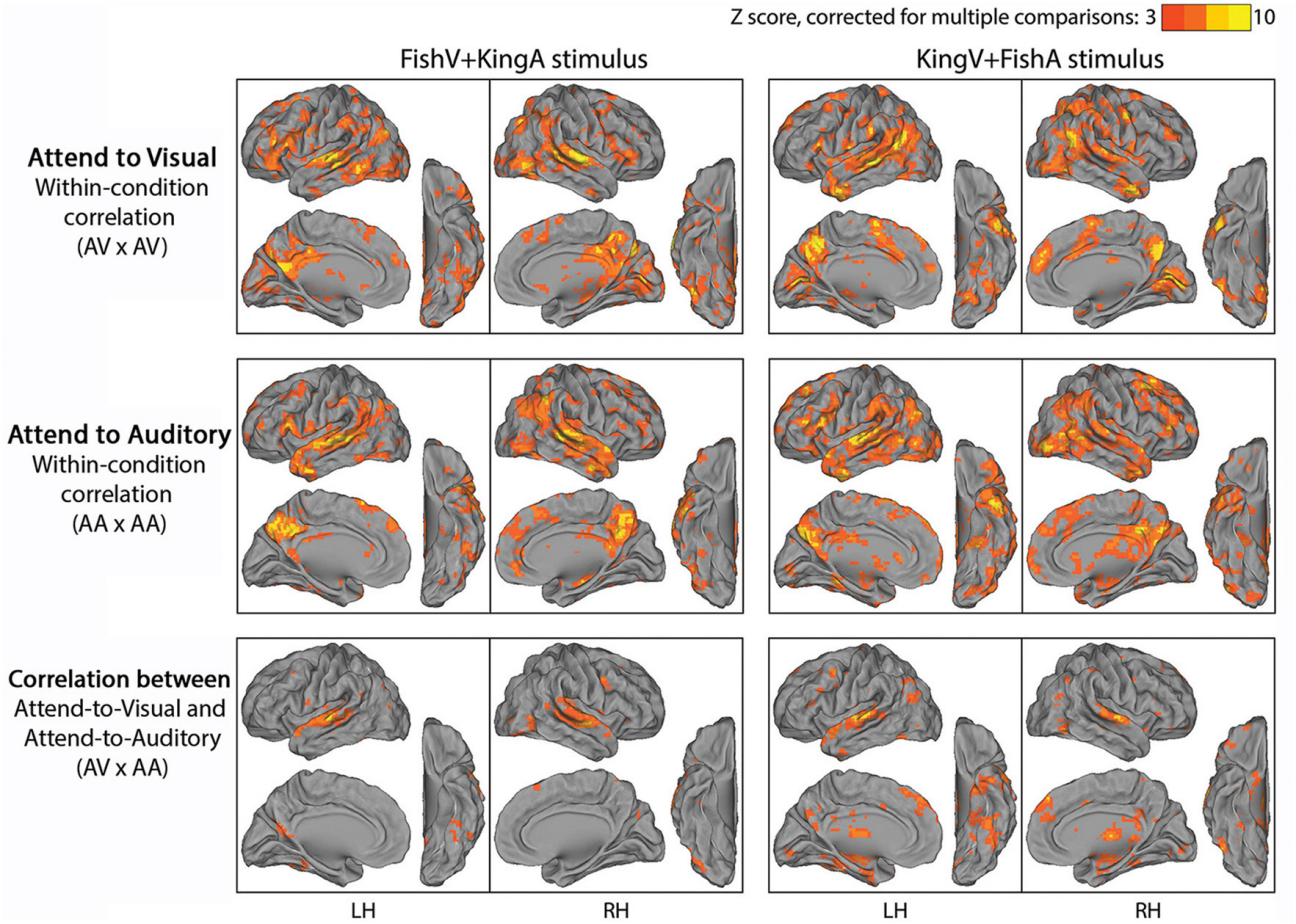
**Results of Experiment 2**. In the top and middle rows, fMRI time series were correlated across subjects under a specific task condition with identical physical stimulus and attentional focus (attending the visual stream in the top row, AV × AV; attending the auditory stream in the middle row, AA × AA). In the bottom row, fMRI time series were correlated across subjects between conditions with identical physical stimulus but opposite attentional focus (AV × AA). Left column, FishV+KingA stimulus; Right column, KingV+FishA stimulus. Population-average correlation values were z-transformed and compared with phase-shuffled data to determine the *Z*-scores (*p* < 0.05, corrected for multiple comparisons).

We first examined which brain regions were consistently modulated by this task. To this end, we correlated fMRI time series across subjects under an identical task condition. Given two stimulus conditions (“FishV+KingA” and “KingV+FishA”) and two attentional states (AV and AA), there were four task conditions in total. Thus, four correlational analyses were carried out between subjects exposed to identical task stimuli and instructions (e.g., between Run 2 from Group 1A and Run 3 from Group 1B). The results from this analysis are shown in Figure 3 (top and middle rows). Widespread brain regions were consistently modulated by this task, with the strongest activities residing in the lateral occipital cortex (LOC), STG, PCC, TP, IFG, and the TPJ.

To locate brain areas involved in the processing of sensory stimulus regardless of attentional focus and the story followed, we correlated fMRI time series between subjects presented with identical physical stimulus but instructed to attend to opposite sensory streams, such that the attended sensory modality (visual vs. auditory) and the story followed (“Fish” vs. “King”) differed between the correlated runs (i.e., Run 2 was correlated between Groups 1A and 1B, and between Groups 2A and 2B). The results of this analysis are shown in the bottom row of Figure 3. Unsurprisingly, auditory cortex along the STG showed reliable responses. In addition, ventral visual areas, thalamus, dmPFC and part of the angular gyrus (AG) were involved. Interestingly, there was limited recruitment of the primary visual cortex (V1), likely because the eye movement pattern differed between the AV and AA conditions, under which the subjects performed active reading and passive fixation, respectively.

**Figure 4:**
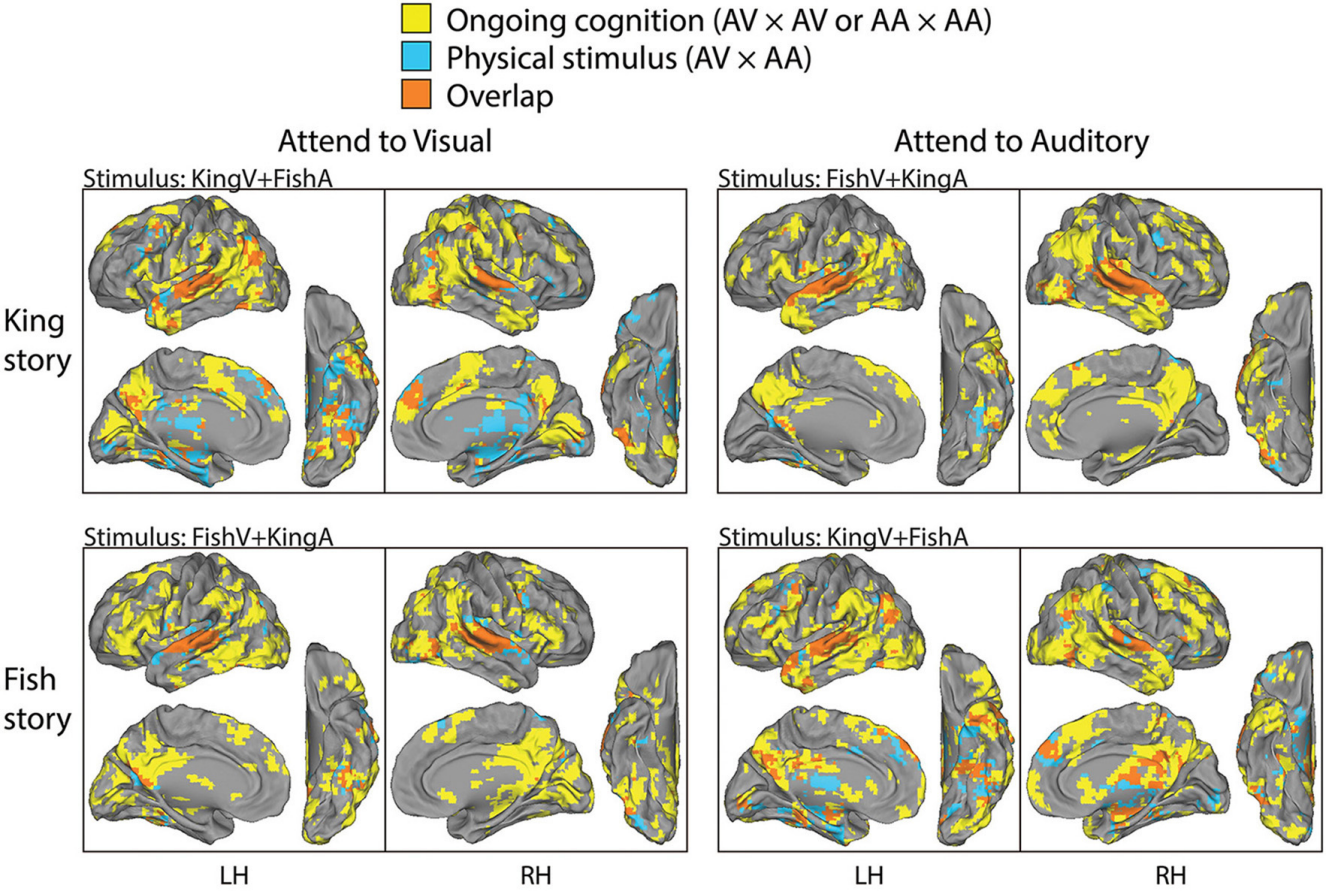
**Parcellation of brain areas modulated by physical stimulus and ongoing cognition**. For each task condition, brain regions consistently modulated by that condition (the significant areas in the top or middle row in Figure 3) are overlaid with those modulated by the physical stimulus used in that condition (significant areas in the bottom row in Figure 3), with the former shown in yellow, the latter in blue, and the overlap between them in orange. Thus, brain areas shown in yellow are those consistently modulated by ongoing cognition beyond the processing of physical sensory stimuli; they form the bases for the analysis described in Figure 5.

The above two analyses respectively extracted brain regions reliably modulated by performing this task (i.e., being exposed to simultaneous auditory and visual streams and attending to one of them) and those reliably modulated by the physical sensory stimuli regardless of the required attentional focus and the story content followed. Hence, contrasting them should reveal brain areas involved in “ongoing cognition” beyond sensory inputs—that is, from attentional fluctuations (if they were similar across subjects) and the understanding of the evolving story content to post-semantic processes such as imagery and emotional response. In Figure 4, we overlaid the results from the first analysis showing regions consistently modulated by performing this task (shown in yellow, from the top two rows in Figure 3) and those from the second analysis showing regions consistently modulated by the physical stimulus alone (shown in blue, from the bottom row in Figure 3), with their overlaps shown in orange. For example, in the top-left panels, yellow/orange regions are those reliably modulated when subjects were presented with the KingV+FishA stimulus and attended to the visual stream; the blue/orange regions were those consistently modulated between the AV and AA conditions under the KingV+FishA stimulus. Thus, regions in yellow represent those contributing to ongoing cognition beyond the processing of physical sensory inputs. Next, we extracted these regions and investigated whether, and if so how, their patterns depended on the attended sensory modality.

**Figure 5:**
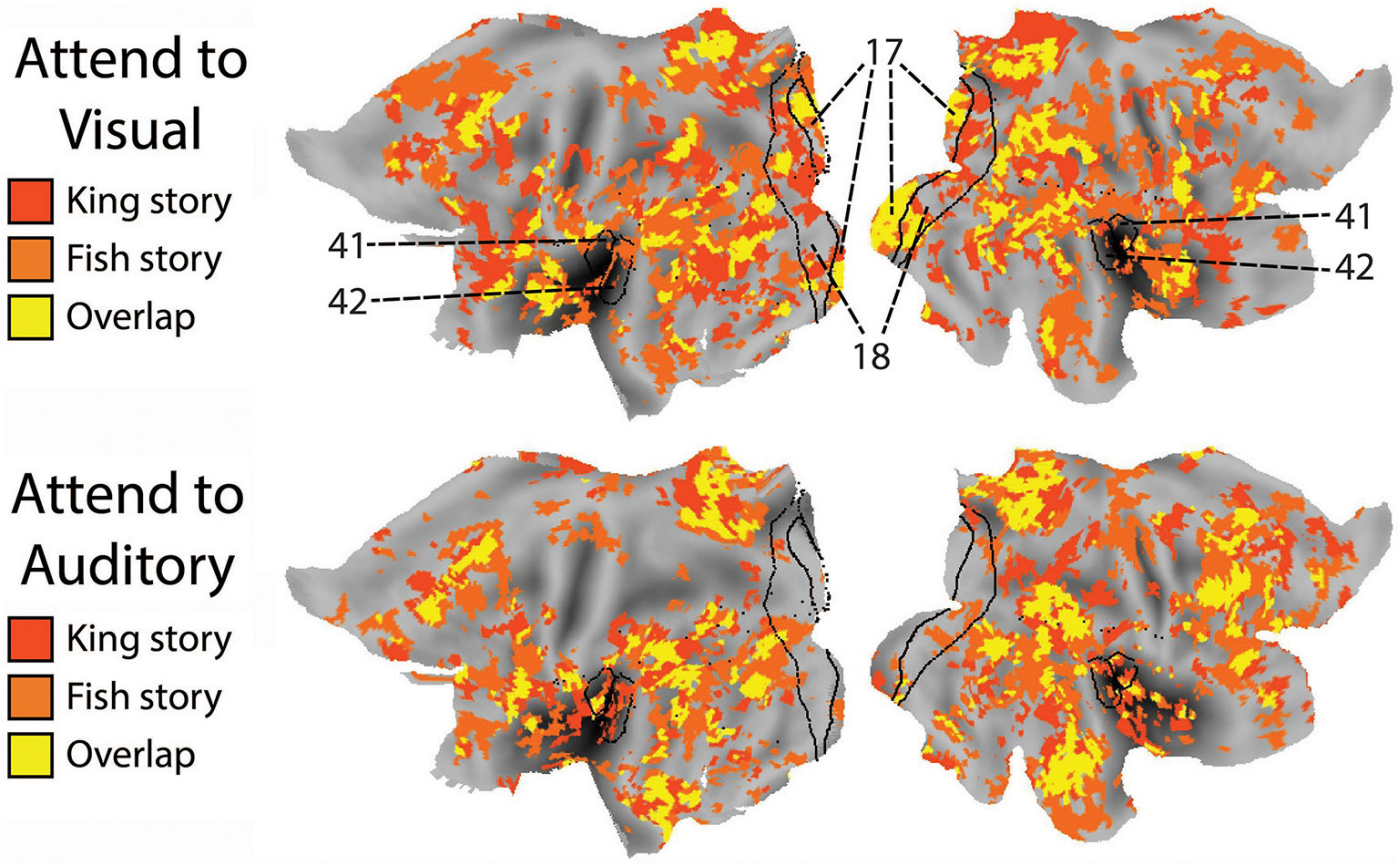
**The effect of attended modality on brain areas contributing to ongoing cognition. Top:** Putative brain areas underlying ongoing cognition identified in the AV condition were combined across the two stories (i.e., the yellow regions in Figure 4, left column). **Bottom:** Putative brain areas underlying ongoing cognition identified in the AA condition were combined across the two stories (i.e., the yellow regions in Figure 4, right column). Black lines mark the borders of primary and secondary visual areas (Brodmann Areas 17 and 18) and the primary auditory cortex (Brodmann Areas 41 and 42).

To compare the putative brain areas involved in ongoing cognition between the AV and AA conditions, we combined the yellow regions in Figure 4 across the “King” and “Fish” stories for the AV (Figure 4, left column) and AA (right column) conditions, respectively. The results are shown in Figure 5. As expected, low-level visual areas (Brodmann areas 17 and 18) were more reliably modulated in the AV condition, and the primary auditory cortex (Brodmann areas 41 and 42) was more reliably modulated in the AA condition. These results suggest that primary sensory areas may also contribute to ongoing cognition, depending on the attentional focus.

## Discussion

In summary, we used an audiovisual cross-modal approach to map brain activity patterns underlying ongoing cognition elicited by hearing or reading a story, and to dissociate these activity patterns from modality-specific sensory processing. The brain areas we identified bear significant similarities to previously described semantic network (Martin, 2007; Patterson et al., 2007; Binder et al., 2009; Schwartz et al., 2011; Honey et al., 2012a; Wei et al., 2012; Fairhall and Caramazza, 2013) and the default-mode network (DMN) (Raichle et al., 2001; Buckner et al., 2008), adding further support for the anatomical convergence of these different brain functions.

In the first experiment, we presented a factual story about the composition of *4′33″* by John Cage to subjects via either the auditory or visual modality. The visual presentation of the story was synchronized phrase by phrase with the auditory version, which comprised of natural reading by a native English speaker. By correlating brain activity fluctuations between subjects who listened to the story and those who read the story, we were able to extract brain regions consistently modulated by this story regardless of the sensory modality used for stimulus delivery. The most consistently modulated brain areas included the STS/STG, the IFG, and the TP (Figure 2), all of which are well-known nodes within the semantic network (Patterson et al., 2007; Binder et al., 2009). Our results from Experiment 1 are largely similar to a previous study that used very similar experimental design (Regev et al., 2013). The main difference between our results and theirs is that we did not find robust cross-modality reliable modulation in PCC (Figure 2B), which is consistent with its lack of reliable modulation within the visual condition alone (Figure 2A, top). This difference might result from the fact that Regev et al. used a real-life story with autobiographical content, which is known to activate the PCC (Buckner et al., 2008), while the “Cage” story we used in Experiment 1 is an encyclopedic entry about a piece of controversial musical history.

In the second experiment, by presenting two stories simultaneously to the subjects via visual and auditory modalities, and cueing them to attend to one or the other in different runs, we were able to manipulate the content of ongoing cognition under identical physical stimulus. This attentional modulation was successful, as demonstrated by the low level of intrusion from the unattended story (rated ~2 on a scale from 1 to 4) and the satisfactory comprehension of the attended story (on average, subjects answered 4.2 and 4.3 out of 5 questions correctly for “King” and “Fish,” respectively). We extracted brain regions consistently modulated by performing this task (by correlating fMRI signals across subjects under an identical task condition), and those modulated by the physical stimuli alone (by correlating fMRI signals between task conditions with identical stimulus but opposite attentional focus). Contrasting these results, we found an extensive brain network consistently modulated by ongoing cognition beyond physical sensory inputs, which had very similar spatial patterns for the two stories (Figure 4). The main regions involved included the TPJ, IFG, TP, PCC, and the posterior middle temporal gyrus (pMTG), consistent with previous studies on semantic processing (Binder et al., 2009; Honey et al., 2012a; Wei et al., 2012; Fairhall and Caramazza, 2013). Nonetheless, there were some subtle differences between the activation patterns in response to the two stories. First, the “Fish” story elicited stronger response in the retrosplenial cortex, potentially due to the strong autobiographical nature of this story. Second, in both hemispheres, the activation pattern around the TPJ was continuous for the “King” story, while it comprised of three disjoint regions in the SMG, AG, and pMTG in response to the “Fish” story. At present the origin of this difference is unclear. One potential contributor is the different levels of theory-of-mind processing involved (Buckner et al., 2008; Corbetta et al., 2008; Carter and Huettel, 2013): In particular, the “King” story, which involves a substantial amount of conjecturing of the character's mental state, evoked an activation pattern around the TPJ that is very similar to previous findings on theory-of-mind (see Figure 12C in Buckner et al., 2008).

Interestingly, the regions identified in Experiment 2 were more extensive than those found in Experiment 1. Two factors may have contributed to this difference: First, the comparative analysis illustrated in Figure 4 may not have completely removed brain regions involved in modality-specific processing. Specifically, since we used conservative whole-brain multiple comparisons correction (*p* < 0.05, FEW corrected) to identify brain areas modulated by the physical stimuli (Figure 3 bottom row, show as blue/orange in Figure 4), there may well be brain areas that we did not have power to detect with this statistical threshold. Second, the difference in story content may have contributed to the more extensive response patterns in Experiment 2: While “Cage” is an encyclopedic entry, “King” and “Fish” are vivid fable and personal stories that are likely to elicit stronger imagery, theory of mind, episodic, and emotional processing.

Many of the brain areas identified in this study as underlying ongoing cognition are well-known components of the DMN, including the PCC, dmPFC, the inferior parietal lobule, and the MTG (Raichle et al., 2001; Fox et al., 2005; Buckner et al., 2008). Previous studies have established that the DMN is activated during “task-independent thought,” suggesting that it may be involved in spontaneous cognition (McGuire et al., 1996; Mason et al., 2007; Christoff et al., 2009). Our results extend these previous findings by showing that the DMN time courses are similarly modulated over time across different individuals by the ongoing cognition evoked by a story regardless of the sensory modality used for story presentation, supporting the idea that the *continuous* activity fluctuations in the DMN are reliably modulated by the evolving flow of conscious content (He and Raichle, 2009).

The similarity between the “semantic network” and the DMN has long been noted (Binder et al., 1999, 2009; Wei et al., 2012; Fairhall and Caramazza, 2013). To account for this observation, Binder et al. (1999, 2009) proposed that semantic processing constitutes a large component of spontaneous thoughts under the resting state, during which the DMN is typically more active. The present study identified brain areas consistently modulated by the evolving content of a story, which relies on semantic processing. Thus, the similarity between the present results and the semantic network as well as the DMN is not surprising. Nonetheless, we believe that potential differences in the spatial patterns among the DMN, the semantic network and the brain areas involved in ongoing cognition should be an interesting and important topic for future research. For example, previous studies found that in addition to the DMN, the executive network including the dorsolateral prefrontal cortex (DLPFC) and the dorsal anterior cingulate cortex (dACC) were also involved in task-independent thoughts (Christoff et al., 2009; Spreng et al., 2010). This is similar to our results (Figures 2, 4). Future studies employing detailed dissection of how the specific content of ongoing cognition or semantic processing relates to specific brain activity patterns would be needed to shed light on the intricate functional/anatomical brain architecture supporting these functions, and how these structures intertwine with the subsystems of the DMN (Andrews-Hanna et al., 2010). A promising approach to this end is demonstrated by a recent study using a data-driven generative model applied to fMRI data obtained under natural movie viewing, which revealed a continuous semantic space across the cortical surface (Huth et al., 2012). As the authors noted in that paper, because only visual stimuli were used in their study, visual and conceptual features were likely mixed in their results. Combining the generative model used therein and the present cross-modal approach in the context of natural stimuli should allow future studies to map the hetero-modal semantic space in the human brain.

As mentioned in Introduction, consistent temporal modulation in the presence of similar conscious stream constitutes a necessary albeit insufficient condition for identifying brain activities underlying the conscious flow. An important future direction is to dissociate brain activities directly contributing to the conscious flow and those consistently modulated by the unconscious processes related to the conscious flow, in line with the recently proposed framework of the tripartite process including prerequisites for the neural correlate of consciousness (NCC-pr), neural correlate of consciousness proper (NCC), and consequences of the neural correlate of consciousness (NCC-co) (Bachmann, 2009; Aru et al., 2012; de Graaf et al., 2012; Li et al., 2014). We anticipate that novel cognitive paradigms and/or analytical approaches will need to be developed in order to separate these processes in the context of ongoing cognition. Lastly, the use of electrophysiological recordings (e.g., Honey et al., 2012b) in the context of these paradigms should shed additional light on the underlying neural mechanisms.

### Conflict of interest statement

The authors declare that the research was conducted in the absence of any commercial or financial relationships that could be construed as a potential conflict of interest.
