# Supplementary material for: A cross-modal investigation of the neural substrates for ongoing cognition
Source: Front Psychol. 2014 Aug 26;5:945. doi: 10.3389/fpsyg.2014.00945 (PMC4143722; doi:10.3389/fpsyg.2014.00945)
Supplement: Supplementary file 4 [file DataSheet1.DOCX]

**“Cage” story**

*4'33"* ("Four minutes, thirty-three seconds") is a three-movement musical composition by American experimental composer John Cage. It was composed in 1952 for any instrument or combination of instruments. The score instructs the performer not to play the instrument during the entire duration of the piece throughout the three movements. The movement durations are, respectively, 30 seconds, 2 minutes and 23 seconds, and 1 minute and 40 seconds. The piece purports to consist of the sounds of the environment that the listeners hear while it is performed, although it is commonly perceived as “four minutes thirty-three seconds of silence.” In a 1982 interview, and on numerous other occasions, Cage stated that *4'33"* was, in his opinion, his most important work.

Silence had already played a major role in several of Cage's works composed before *4'33"*. The *Duet for Two Flutes*, composed when Cage was 22, opens with silence. In his songs *The Wonderful Widow* *of Eighteen Springs* and *A Flower*, Cage directs the pianist to play a closed instrument, which may be understood as a metaphor of silence.

In 1951, Cage visited the anechoic chamber at Harvard University. An anechoic chamber is a room designed in such a way that the walls, ceiling, and floor absorb all sounds made in the room, rather than reflecting them as echoes. Such a chamber is also externally sound-proofed. Cage entered the chamber expecting to hear silence, but he wrote later, “I heard two sounds, one high and one low. When I described them to the engineer in charge, he informed me that the high one was my nervous system in operation, the low one, my blood in circulation.” Cage had gone to a place where he expected total silence, and yet heard sound. “Until I die there will be sounds. And they will continue following my death. One need not fear about the future of music.” The realization of the impossibility of silence led to the composition of *4'33"*.

Another influence for this piece came from the field of the visual arts. Cage's friend and sometimes colleague Robert Rauschenberg had produced a series of white paintings. These seemingly blank canvases in fact change according to varying light conditions in the rooms in which they are hung, the shadows of people in the room, and so on. This inspired Cage, as he later stated, “Actually what pushed me into it was not guts but the example of Robert Rauschenberg. His white paintings... when I saw those I said ‘Oh yes, I must. Otherwise I'm lagging, otherwise music is lagging.’” Cage's musical equivalent to the Rauschenberg paintings uses the “silence” of the piece as an aural “blank canvas” to reflect the dynamic flux of ambient sounds surrounding each performance. The music of the piece comes from the sounds of the players, the audience, the building, and the outside environment.

*4'33"* is an example of automatism, in which the composers wish to completely remove themselves from the process of creation. This is motivated by the belief that creation without social pressure is impossible, as there is no way for individuals to truly express themselves without infusing into the art the social standards that they have been subjected to since birth. Therefore, the only way to achieve truth is to remove the artist from the process of creation. In *4'33"*, Cage has no way of controlling what the audience will experience.

His composition challenges, or rather exploits to an extent, the social regiments of the modern concert life etiquette, experimenting on unsuspecting concert-goers to prove three important points. First, the choice of a prestigious venue and the social status of the composer and the performers naturally heightens the audience's expectations for the piece. As a result, the listener is more focused, giving Cage's *4'33"* the same amount of attention as if it were Beethoven's *9th*. Thus, even before the performance, the reception of the work is already predetermined by the social setup of the concert. Furthermore, the audience's behavior is limited by the rules of the concert hall. It is not easy to get a large group of people to listen to ambient noise for nearly five minutes unless they are regulated by the concert hall etiquette.

The second point made by *4'33"* concerns duration. According to Cage, duration is the essential building block of all music. Each duration of time can be filled with either sounds, silence, or noise, but no single one of these elements is absolutely necessary for completeness. Cage managed to emancipate the silence and the noise to make it acceptable or perhaps even an integral part of his composition. This piece serves as a radical and extreme illustration of this concept.

The third point is that the work of music is defined not only by its content but also by the behavior it elicits from the audience. In Cage's *4'33"*, the audience may feel cheated by having to listen to no composed sounds from the performers. Nevertheless, the audience contributes the bulk of the musical material. Since the piece consists of exclusively ambient noise, the audience's behavior, their whispers and movements, are essential elements. John Cage's *4'33"* remains controversial to this day, and is seen as challenging the very definition of music.

**“King” story**

It once occurred to a certain king, that if he always knew the right time to begin everything; if he knew who were the right people to listen to, and whom to avoid; and, above all, if he always knew what was the most important thing to do, he would never fail in anything he might undertake. He decided to consult a hermit, widely renowned for his wisdom.

The hermit lived in a wood which he never quitted, and he received none but common folk. So the King put on simple clothes, and before reaching the hermit's cell, dismounted from his horse, and, leaving his body-guard behind, went on alone. When the King approached, the hermit was digging the ground in front of his hut. Seeing the King, he greeted him and went on digging. The hermit was frail and weak, and each time he stuck his spade into the ground and turned a little earth, he breathed heavily. The King went up to him and said: “I have come to you, wise hermit, to ask you to answer three questions: How can I learn to do the right thing at the right time? Who are the people I most need, and to whom should I, therefore, pay more attention than to the rest? And what affairs are the most important, and need my first attention?” The hermit listened to the King, but answered nothing. He just spat on his hand and recommenced digging. “You are tired,” said the King, “let me take the spade and work awhile for you." One hour passed, and another. The sun began to sink behind the trees, and the King at last stuck the spade into the ground, and said: “I came to you, wise man, for an answer to my questions. If you can give me none, tell me so, and I will return home.”

“Here comes someone running,” said the hermit, “let us see who it is.” The King turned round, and saw a bearded man come running out of the wood. The man held his hands pressed against his stomach, and blood was flowing from under them. When he reached the King, he fell fainting on the ground moaning feebly. The King and the hermit unfastened the man's clothing. There was a large wound in his stomach. The King washed it as best he could, and bandaged it with his handkerchief and with a towel the hermit had. Meanwhile the sun had set, and it had become cool. So the King, with the hermit's help, carried the wounded man into the hut and laid him on the bed. The King was so tired with his walk and with the work he had done, that he crouched down on the threshold, and also fell asleep.

When he awoke in the morning it was long before he could remember where he was, or who was the strange bearded man lying on the bed. “Forgive me!” said the bearded man in a weak voice, when he saw that the King was awake and was looking at him. “I do not know you, and have nothing to forgive you for,” said the King. “You do not know me, but I know you. I am that enemy of yours who swore to revenge himself on you, because you executed his brother and seized his property. I knew you had gone alone to see the hermit, and I resolved to kill you on your way back. But the day passed and you did not return. So I came out from my ambush to find you, and I came upon your bodyguard, and they recognized me and wounded me. I escaped from them, but should have bled to death had you not dressed my wound. I wished to kill you, and you have saved my life. Now, if I live, and if you wish it, I will serve you as your most faithful slave, and will bid my sons to do the same. Forgive me!”

The King was very glad to have made peace with his enemy so easily, and to have gained him for a friend, and he not only forgave him, but said he would send his servants and his own physician to attend him, and promised to restore his property. Having taken leave of the wounded man, the King went out into the porch and looked around for the hermit. Before going away he wished once more to beg an answer to the questions he had put. The hermit was outside, on his knees, sowing seeds in the beds that had been dug the day before. The King approached him, and said: “For the last time, I pray you to answer my questions, wise man.” “You have already been answered!” said the hermit. “How answered? What do you mean?” asked the King.

“Do you not see,” replied the hermit. “If you had not pitied my weakness yesterday, and had not dug those beds for me, but had gone your way, that man would have attacked you, and you would have repented of not having stayed with me. So the most important time was when you were digging the beds; and I was the most important man; and to do me good was your most important business. Afterwards when that man ran to us, the most important time was when you were attending to him, for if you had not bound up his wounds he would have died without having made peace with you. So he was the most important man, and what you did for him was your most important business. Remember then: there is only one time that is important-- Now! It is the most important time because it is the only time when we have any power. The most necessary man is he with whom you are, for no man knows whether he will ever have dealings with anyone else: and the most important affair is, to do him good, because for that purpose alone was man sent into this life!”

**“Fish” story**

I am Fred's fish. As to what I did to offend the Gods above to deserve such a fate is beyond my comprehension. You see, I don't mind being a fish; rather enjoy it, as a matter of fact. But it's Fred. To put it bluntly, the man's a putz. He's 37 years old and just moved out of his mother's house last summer. He enters logarithmic data for some computer corporation and writes romance novels on the side. We live in an upstairs, modest, big-city apartment, and as you could probably guess, the guy hasn't had a date in as long as I've known him, which is no small amount of time. I mean, not to complain, he's a nice guy and all, keeps the place super clean, but I just think he needs to get out more. His problem is that he's too overprotective of me. Hey, I'm a grown fish; I can take care of myself!

For example, the other day he was in one of his usual melancholy moods and pulled up a chair next to my bowl and just stared at me. Well a long time ago, Fred was mad at me and tried to stare me down, just to let me know who the boss was around here. But little did he know, fish don't have eyelids, and him so set on making me blink first sort of dried out his eyeballs. I must say, I felt kind of sorry for him.

Anyways, I thought he was going for one of those deals again, when a wavering tear slowly rolled down his cheek and in a choked-up voice, began talking to me. “Mr. Fish, you are my best friend. I just want you to know that I will do everything in my power to protect you and keep you happy. If for some reason, the Earth should implode into an empyreal fireball and your glass bowl disintegrate, I will dive across the floor and catch you and hold you under a faucet until help comes. I just want to thank you for always being here when I need you.” Wow. Empyreal fireball. The guy talks to me on a regular basis, usually telling me his troubles, but never with so much emotion. I just stared at him and he threw his arms around my bowl and we sat there in an embrace for quite a while. It was pretty embarrassing.

So there's the gist of our relationship. He feeds me around 7 o'clock in the A.M. and goes off to work. I putter around the bowl for a while, keeping myself occupied. Ponder on life's great mysteries. Like why people make fluorescent sand. Did I mention I hate that stuff? Of course that's what Fred puts at the bottom of my bowl, no matter how much I protest. It's so tacky. He comes home at night, tells me about his day at work and the usual antics of his co-workers, and then writes for a while. Around 8 o'clock he feeds me again and moves my bowl in front of the T.V. so we can watch sitcom reruns. On Saturdays the dip brings me around to watch the weekly fishing channel with him, of all things. Is this man a shmuck or what? Does he really believe that I want to watch ogre men catch my grandmother or cousins and turn them into a pâté? Even if I wasn't a fish, I'd still be disturbed by this fact. I mean, doesn't Fred have anything better to do on Saturdays than watch... the fishing channel? I do have my ways of getting back at him though. Just for a laugh, I roll over on my back and float to the top of my bowl. Boy, does Fred scream and cry! His eyes bulge out and he flies over to me. Just when I think he's going to try mouth-to-mouth I roll back over and swim to the bottom. Ahh, it at least gives a change from the usual routine.

Fred isn't completely friendless. Well, maybe he is now, I don't know. But up until about a month ago, this guy Dave would come around every now and again. I liked Dave. He was pretty wild and crazy. But last month when he was over, him and Fred were drinking what they called “brewskis” and laughing up a storm. Dave came over to me and poured a can of it into my bowl, saying, “I wonder if fishy can party with the boys?!” I didn't know what to make of it. It tasted good, and provided a change from my usual fish flakes. But Fred got suddenly angry. I had never seen him this mad. He screamed at Dave to leave me alone and kicked him out of the apartment. He changed my water immediately and started apologizing to me. I was now feeling pretty good for some reason, and just wanted to laugh at him. In fact, I did laugh at him. A great big, bellowing laugh as only fish can laugh. But suddenly I started to feel dizzy and sick. Now I was floating at the top of my bowl and this time I wasn't faking it. Fred just cried and told me I would soon be okay. He was right. The next day I was back to my frisky old self. What a story for the grandkids!

So anyways, there's my life. Barring any unforeseen incidents, I guess it's pretty good to be where I am. Now that I really think of it, you can't ask for a better caretaker than Fred. I sometimes wish I could be Fred, just for a day or so, and Fred often confides in me that he wishes he could be a fish. To be taken care of, live free and without worries. Well, I don't know about being free, but all-in-all it's a good life and I can't think of anything else I'd rather be than Fred's fish.
